# Solid Plasmonic Substrates for Breast Cancer Detection by Means of SERS Analysis of Blood Plasma

**DOI:** 10.3390/nano10061212

**Published:** 2020-06-21

**Authors:** Gabriela Fabiola Știufiuc, Valentin Toma, Mihail Buse, Radu Mărginean, Gabriela Morar-Bolba, Bogdan Culic, Romulus Tetean, Nicolae Leopold, Ioana Pavel, Constantin Mihai Lucaciu, Rareș Ionuț Știufiuc

**Affiliations:** 1 Faculty of Physics, “Babeș-Bolyai” University, M. Kogalniceanu 1, 400084 Cluj-Napoca, Romania; gabi.stiufiuc@phys.ubbcluj.ro (G.F.Ș.); romulus.tetean@phys.ubbcluj.ro (R.T.); nicolae.leopold@phys.ubbcluj.ro (N.L.); 2 MedFuture Research Center for Advance Medicine, “Iuliu Hațieganu” University of Medicine and Pharmacy, L. Pasteur 4-6, 400349 Cluj-Napoca, Romania; valentin.toma@umfcluj.ro (V.T.); buse.mihail@umfcluj.ro (M.B.); margi.radu@outlook.com (R.M.); 3 Department of Senology, The Oncology Institute “Prof. Dr. Ion Chiricuta”, 34-36 Republicii Street, 400015 Cluj-Napoca, Romania; gabrielambolba@yahoo.co.uk; 4 Department of Dental Propedeutics and Esthetics, “Iuliu Hațieganu” University of Medicine and Pharmacy, 8 V. Babes Street, 400012 Cluj-Napoca, Romania; bculic@umfcluj.ro; 5 Department of Chemistry, Wright State University, 3640 Colonel Glenn Hwy., Dayton, OH 45435-0001, USA; ioana.pavel@wright.edu; 6 Department of Pharmaceutical Physics-Biophysics, “Iuliu Hațieganu” University of Medicine and Pharmacy, L. Pasteur 6, 400349 Cluj-Napoca, Romania

**Keywords:** SERS, solid plasmonic substrates, biofluids, MVA-SERS based cancer detection

## Abstract

Surface enhanced Raman spectroscopy (SERS) represents a promising technique in providing specific molecular information that could have a major impact in biomedical applications, such as early cancer detection. SERS requires the presence of a suitable plasmonic substrate that can generate enhanced and reproducible diagnostic relevant spectra. In this paper, we propose a new approach for the synthesis of such a substrate, by using concentrated silver nanoparticles purified using the Tangential Flow Filtration method. The capacity of our substrates to generate reproducible and enhanced Raman signals, in a manner that can allow cancer detection by means of Multivariate Analysis (MVA) of Surface Enhanced Raman (SER) spectra, has been tested on blood plasma samples collected from 35 healthy donors and 29 breast cancer patients. All the spectra were analyzed by a combined Principal Component-Linear Discriminant Analysis. Our results facilitated the discrimination between healthy donors and breast cancer patients with 90% sensitivity, 89% specificity and 89% accuracy. This is a direct consequence of substrates’ ability to generate diagnostic relevant spectral information by performing SERS measurements on pristine blood plasma samples. Our results suggest that this type of solid substrate could be employed for the detection of other types of cancer or other diseases by means of MVA-SERS procedure.

## 1. Introduction

Nowadays, screening for known cancer biomarkers is considered to be one of the most successful strategies for cancer prevention, diagnosis and/or improvement of survival rate [1,2], as opposed to traditional cancer screening methods which do not consistently provide the desirable results. For instance, in the case of breast cancer screening by means of mammography, there is little or no evidence that it contributes to reduced mortality rate [3]. An explanation for this unexpected outcome arises due to the fact that mammography screening has led to a significant increase in over-diagnosis, while the number of advanced breast cancer patients discovered through mammography remained practically unchanged [4,5]. As breast cancer (BC) is the leading cause of death related to neoplastic disease in women worldwide [6], an alternative screening method with substantial benefits is urgently needed. Furthermore, this challenge exists for all types of cancer, but according to recent reports [7] there is a strong belief that surface enhanced Raman spectroscopy (SERS)-based strategies could bring a major improvement in this area, that is beneficial, not only for researchers, but most importantly for patients. Given that blood tests are routinely used to monitor health condition [8], an ideal solution for the issue might be introducing a simple, time and cost-effective blood test that could accurately discriminate between healthy and diseased patients in a very reproducible manner [9]. The three aforementioned criteria are often met by optical analytical approaches, out of which vibrational spectroscopy techniques have the greatest advantage in providing “molecular fingerprints” of complex biological mixtures with no need for extrinsic labeling [10].

Raman spectroscopy is particularly desirable from this point of view, as it is not limited by water contributions, sequentially emerging as a powerful analytical method that allows for the examination of a great variety of samples. A hot topic regarding the use of this technique is in the analysis of biological samples, including fluids (e.g., blood and derivatives, saliva, urine), cells and tissue [10,11]. Amongst these, blood and its derivatives represent the richest source of vibrational information regarding physiological and pathological processes that take place all over the organism, particularly due to the fact that it circulates through all body structures. Raman spectroscopy has proven to be a valuable analytical tool in bio-detection and molecular mapping studies, and subsequently new exciting applications in the field of cancer diagnosis and prognosis are emerging [12,13]. Unfortunately, this technique possesses quite a low sensitivity and can be often accompanied by a high fluorescence background. These drawbacks restrain its applicability in the analysis of low concentration samples such as the ones obtained from bio-fluids [11,13,14]. In principle, these limitations can be overcome by means of several derived techniques, such as surface enhanced Raman spectroscopy (SERS) or resonant Raman spectroscopy (RRS). SERS has the advantage of fluorescence quenching, a significantly higher sensitivity and a spatial resolution, lower than the diffraction limit of white light optical microscopic techniques. Its use in biological applications encounters several issues related to the non-specific interaction of the plasmonic substrates, with highly complex biological samples, especially blood and its derivatives. One of the biggest problems that needs to be overcome is the random attachment of proteins (the main molecular constituents of blood plasma and serum) to the SERS substrates. This phenomenon leads to a full coverage of the active sites, meaning that other molecules of interest cannot reach these spots [14]. So far, several strategies have been developed for obtaining a reproducible SERS fingerprint of blood samples. Bonifacio et al. have demonstrated that the fastest and most efficient method of rapidly collecting Surface Enhanced Raman (SER) spectra is after deproteinization of the blood serum [15]. The analysis of the experimental SER spectra allowed them to separate cancer patients from healthy subjects [16]. However, the most important requirement consists in obtaining plasmonic substrates capable of generating consistent and scientifically relevant SER spectra [17].

Over the years our group has developed several original synthesis methods for silver [18] and gold [19,20,21,22] nanoparticles (NPs). They possess interesting plasmonic properties allowing the detection of analytes at low concentrations or even chiral discrimination by means of SERS [23,24]. Herein, we propose a straightforward synthesis method for solid SERS-active substrates, based on silver colloids that were purified and concentrated via the tangential flow filtration (TFF) technique. The proposed solid plasmonic substrates facilitate the generation of reproducible SER spectra in the case of blood plasma samples collected from a statistical relevant number of donors (*n* = 35) and BC patients (*n* = 29), even when using an excitation wavelength from the NIR region (785 nm). A careful examination of these spectra revealed that plasmatic small biomolecules contribute significantly to large proteins, probably due to their higher affinity for this type of solid silver plasmonic substrate (SSPS). As such, the first major goal of this study was to prove that a successful SERS-based cancer detection method begins with the production of a suitable plasmonic substrate. In the case of SERS measurements on biofluids, this substrate has to be able to generate reproducible and “diagnostic relevant” vibrational spectra that will be further analyzed by means of Multivariate Analysis.

By performing a combined Principal Component Analysis-Linear Discriminant Analysis (PCA-LDA) of the experimental SER spectra recorded on our solid substrates we were able to discriminate with high sensitivity, specificity and accuracy (90% sensitivity, 89% specificity, 89% accuracy) the healthy donors’ group from BC patients’ group.

The obtained results emphasize the major role played by the plasmonic substrates in SERS measurements performed on blood plasma and open the possibility for using the here presented solid substrates for SERS-based detection of other types of cancer. Nevertheless, it should be noted that the purpose of this article is not to not to characterize the biological relevancy of BC or its detection. This will be addressed in future research. We emphasize the importance of the substrate towards SERS analysis as we believe it is the best way to attain reproducible and diagnostic relevant results.

## 2. Materials and Methods

All the chemicals utilized in this study were of analytical grade and used just as it is from the producer company, without further purification. More specifically, the silver nitrate (AgNO_3_) and hydroxylamine (NH_2_OH) were purchased from Roth GmbH (Gladbach, Germany). The subsequent aqueous solutions explicitly utilized in the experimental procedures were prepared using ultrapure water (18.2 MΩ × cm, ELGA Labwater from PURELAB Chorus, Buckinghamshire, UK). Lastly, the synthesis of solid plasmonic SERS substrates entailed using CaF_2_ polished glasses (20 mm diameter and 1 mm thickness, purchased from Crystran Limited, Poole, UK) as port-probes for.

### 2.1. Synthesis of Silver Colloids

The procedure proposed by Leopold and Lendl was used to synthesize the hydroxylamine reduced silver colloids (AgHya) [25]. A total of 90 mL of ultra-purified water was used to dissolve 10.5 mg of hydroxylamine hydrochloride and 12 mg of sodium hydroxide in a Simax ISO blue capped glass bottle of 250 mL. In a second bottle of the same making, 17 mg of silver nitrate was dissolved in 10 mL of ultra-purified water. Under vigorous stirring conditions, this solution was promptly poured in the previous one. It should be noted, that if the protocol is followed correctly, the final solution should quickly change color from colorless to brown and finally to yellowish grey over the course of 5 min. Once the synthesis process completed, tangential flow filtration (TFF) technique was implemented for the purification and concentration of the NPs, as described in the literature [26,27]. This step turned out to have a major role in the synthesis of suitable substrates for SERS measurements performed on bio-fluids. Firstly, it removes the chemical byproducts resulting from the synthesis procedure allowing a better interaction of the small biomolecules with the silver substrate. Secondly, this procedure reduces the dimensional polydispersity of the AgHya NPs, favoring the reproducibility of the SER spectra.

### 2.2. Preparation of the Plasmonic Solid Substrates

The TFF-processed colloidal solutions were subsequently used as the foundational building blocks in the synthesis of solid plasmonic substrates. First, the CaF_2_ glass were prepared by cleansing with acetone and ethanol, rinsing with ultrapure water and left to air dry. After 15 min, a plate heater was used to heat the port-probe at 40 °C. In the final step, a 1 µL volume of concentrated colloid was pipetted on the CaF_2_ port-probe and left to dry 80 s. The as-obtained SERS solid substrates were removed from the heated plate and cooled down to room temperature; in this state, they ready for use.

### 2.3. Analyte Deposition for SERS Measurements

A 1 µL volume of blood plasma was pipetted on the dry, solid substrates. The deposited analytes were left to air dry inside a refrigerator (~4 °C) for 1 h before proceeding with the SERS measurements.

### 2.4. UV-VIS Absorption Measurements

A T92+ UV-VIS Spectrophotometer from PG Instruments Ltd. (Leicestershire, UK) was used to record the UV-VIS absorption spectra. The absorption curves were acquired on standard quartz cells at room temperature in the 300–800 nm spectral range. Lastly, the spectral resolution was set at 2 nm.

### 2.5. TEM and EDS Measurements

The HT7700 (Hitachi Ltd., Tokyo, Japan) Transmission Electron Microscope (TEM) was used to execute the electron microscopy measurements and the Energy Dispersive Spectroscopy (EDS), operating at 100 kV and using the high-resolution operation mode (spot size 3 = 0.60 µm). The samples were deposited on carbon films on top of copper grids for 2–5 min; the time is dependent on sample type and concentration. After deposition, the excess solution was blotted away using No. 42 ashless filter paper and the grids were left covered at room temperature to completely dry. The obtained images were calibrated for size, annotated, and processed (contrast enhancement by histogram stretching, smoothing (sigma = 2.2) and cropping) using the ImageJ software (National Institutes of Health, Bethesda, MD, USA).

### 2.6. Raman and SERS Measurements

The Raman and SERS spectra were measured using the Renishaw™ *inVia* Reflex Raman (Renishaw plc, Gloucestershire, UK) confocal multilaser spectrometer at a resolution of ~1 cm^−^^1^. An internal silicon reference was used for calibration. The SERS/Raman measurements were achieved using small amounts (~1 µL) of analytes; these analyte droplets were pipetted and left to dry, either on solid plasmonic substrates, or directly on CaF_2_ glass. The 50× (N.A = 0.75) objective lens was used to record the spectra. A 785 nm diode laser was used for excitation. The laser power (measurements relative to the sample surface) was less than 65 mW, while the acquisition time was set to 10 s. The spectrograph was equipped with a 1200 lines/mm grating and a charge coupled device camera (CCD). Baseline correction was applied to all SERS spectra, in order to eliminate the fluorescence background. Each spectrum represents the average of minimum 30 spectral acquisitions, collected on different randomly chosen regions of the substrate. The baseline correction was performed by using the Wire 4.2 software provided by Renishaw (Gloucestershire, UK), with the *inVia* spectrometer.

### 2.7. AFM Measurements

Using the NT-MDT NTegra Vita system (NT-MDT Spectrum Instruments, Zelenograd, Russia) mounted on an inverted Olympus IX73 optical microscope, atomic force microscopy (AFM) experiments were performed under ambient conditions. The measurements were done in semi-contact mode, using Si_3_N_3_ tips having a resonant frequency of 235 kHz and a force constant of 12 N/m. The typical curvature radius of the tips is ~10 nm. The images captured different regions of the solid plasmonic substrates using the topographic and phase contrast mode.

### 2.8. NTA Measurements

Nanoparticle Tracking Analysis (NTA) measurements were done using a ZetaView^®^ NTA-Nanoparticle Tracking Video Microscope PMX-120 (Particle Metrix GmbH, Inning am Ammersee, Germany). A 1000 times dilution was done for all colloids with ultra-purified water, before injecting the samples in the device. The recording parameters were automatically set before each analysis.

### 2.9. Research Ethics

All subjects gave their informed consent for inclusion before they participated in the study. The study was conducted in accordance with the Declaration of Helsinki 2013, and the protocol was approved by “Iuliu Hatieganu” University’s Ethics Committee. The reference number for this approval is 206/16.05.2017.

### 2.10. Cohort of Patient Samples

A number of 29 female BC patients and 35 healthy donors were enrolled in the study, as previously mentioned, with informed consent. The samples for the breast cancer (BC) patients were collected immediately after the cancer diagnosis and, therefore, no therapy or treatment interfered with our analyte for the measurements. The patient ages ranged between 32 and 77 years with a median of 54.91 years. In the case of the healthy donors, the ages ranged between 35 and 55 years with a median of 51.54 years. Among the BC patients, the molecular subtypes measured were; 22 luminal B, 6 luminal A, 5 triple negative and one luminal HER2 positive, with stages between I and IV.

### 2.11. Blood Plasma Collection

The blood samples were obtained from the antecubital vein of healthy donors and BC patients using Becton Dickinson vacutainers containing citrate. Immediately after collection, blood tubes were centrifugated at 2600× *g* for 10 min in order to separate the plasma from the rest of the blood. Once the separation procedure finished, the blood plasma was removed from the tube and stored at −80 °C until the measurements were performed.

### 2.12. Multivariate Analysis

Python software (Python Software Foundation, Wilmington, NC, USA) was used to execute a multivariate analysis of the experimental SER spectra. Before being analyzed, all the experimental data have been baseline corrected using the Wire software (Wire Swiss GmbH Zug, Switzerland). Data preprocessing includes spectral normalization by mean intensity. Further predictive analysis was performed using the Pandas package, where Principal Component Analysis (PCA) combined with Linear Discriminant Analysis (LDA) due to their Scikit-learn implementation.

## 3. Results and Discussions

**Figure 1:**
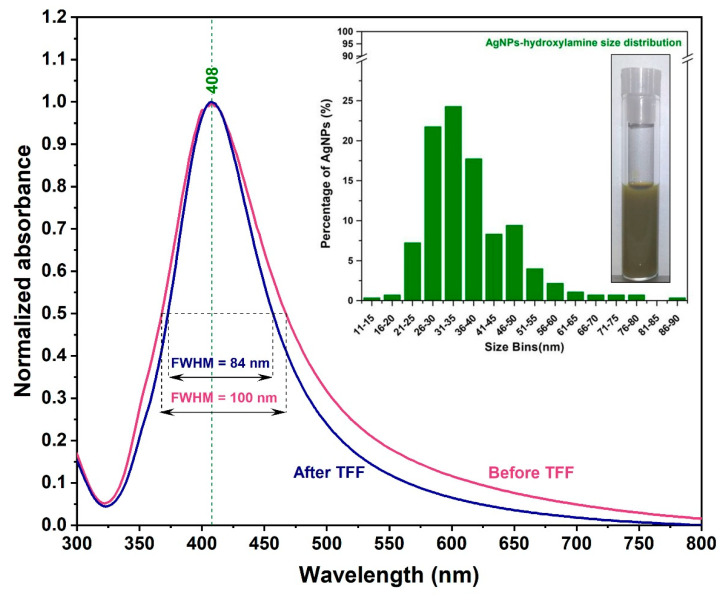
Superposition of the normalized UV-VIS absorption spectra of AgHya colloid recorded before (red curve) and after (blue curve) purification. The optical image of the purified colloid together with the statistical analysis of their dimensions are shown in the inset.

The first major goal of this study was the synthesis of solid plasmonic substrates using as building blocks spherical hydroxylamine reduced silver NPs suitable for SERS measurements on bio-fluids. Once the synthesis procedure completed, the colloidal solutions were purified and concentrated by means of TFF method, which turned out to be a critical step for the use of the currently-proposed solid substrates for SERS measurements on blood plasma. As previously shown, TFF is an alternative technique for purification, concentration, and size-selection of polydisperse colloids [26,27,28]. By removing the chemical by-products produced during the synthesis process, the TFF allowed a better interaction of the small plasmatic biomolecules with the plasmonic substrate. On the other hand, the TFF purification improved the dimensional polydispersity of the AgHya NPs as it has been observed from the UV-VIS absorption measurements perform before and after colloidal purification (Figure 1). At a careful examination of the two absorbance spectra one can observe a 16% reduction of the Full Width Half Maximum (FWHM) value after the purification (100 nm versus 84 nm). This could explain the high degree of reproducibility of the SER spectra recorded on plasma samples, which are presented in this study. TEM measurements (performed on purified samples) confirmed the successful synthesis and purification of quasi-spherical silver NPs, as illustrated in Figure S1. The statistical analysis, performed on a large number of NPs from the TEM images, indicated a mean diameter value of ~33 nm and a high degree of mono-dispersity (inset Figure 1). The statistical analysis has been performed on more than 200 NPs.

These values are in good correlation with the UV-VIS absorption measurements, as the colloid exhibits a narrower, well-defined surface plasmon resonance band and low extinction values at high wavelengths after the purification. NTA measurements re-confirmed the previous results and provided additional information: NP’s concentration has a value of ~4 × 10^10^ NPs/mL. NPs composition has been also confirmed by means of Energy Dispersive Spectroscopy (EDS) technique (Figure S2).

**Figure 2:**
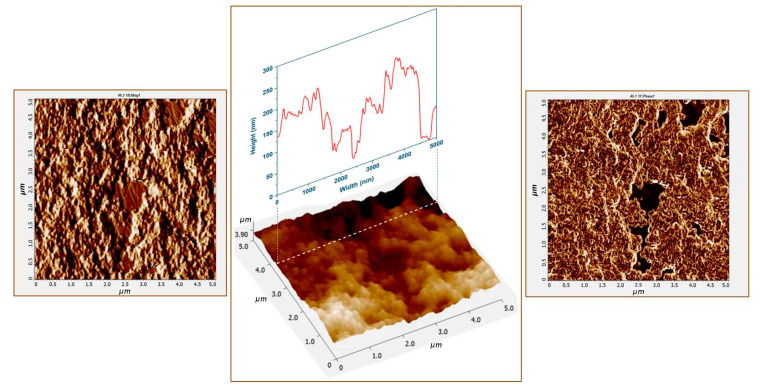
5 × 5 µm^2^ topographic images of the silver solid substrate acquired in magnitude mode (left) and height mode (center). The phase image of the same area is presented on the right image. A height profile is highlighted in the inset of the central image.

Once purified and concentrated, the silver NPs were poured on top of a CaF_2_ substrate heated at 40 °C and let dry for 80 s. A typical atomic force microscopy (AFM) image of the solid substrate formed by heat induced NPs self-assembling is presented in Figure 2. The AFM image has been taken in a region that contains an uncovered zone of CaF_2_ glass that has been used as reference. The height profile (presented in the inset) indicates that the mean height of the substrate (with respect to the glass) has a value of ~120 nm. In the highest points of the substrate it does not exceed 200 nm. By taking into account, the mean value of AgHya NPs diameter of ~33 nm (as deduced from TEM images statistical analysis) one can conclude that the substrate contains several layers of AgHya NPs self-assembled in a three-dimensional (3D) manner onto the glass substrate.

The phase image indicates that material composition is quite homogeneous in the regions where the NPs are present, arguably due to the tangential flow filter (TFF) purification step that was performed prior to substrate formation. It has been shown that AFM phase images can be successfully employed for proving the presence of molecular species on the edges of anisotropic gold NPs [19]. By reducing the number of the molecules present in the very close vicinity of the substrate, the diagnostic relevant small analyte biomolecules, which are found in very small concentrations in the biofluids, are “allowed” to occupy the plasmonic active sites. In this way, their contribution to the vibrational spectrum can be considerably enhanced. This is the reason we tested the capacity of the proposed solid substrate for SERS-based BC detection, based on Multivariate Analysis (MVA) of vibrational spectra, and recorded on blood plasma samples of BC patients and controls.

The enhancement capabilities of the plasmonic substrate have been tested firstly on methylene blue (MB), which is a standard analyte in SERS. The spectra were recorded on a large area (60 × 60 μm^2^) area and the quality of the recorded spectra was found to be quite remarkable. A typical SER spectrum of MB is presented in Figure S3. The second tested analyte was rhodamine 6G (R6G). This analyte was used in a first instance for testing the reproducibility of the recorded spectra and it was also used for the calculation of substrate’s enhancement factor (EF). In a typical procedure, 1μL solution of 10^−3^ M R6G was poured onto the substrate and left to dry. Sixteen spectra were collected on a 60 × 60 μm^2^ substrate area. An optical image of the substrate covered with R6G dried analyte is presented in Figure S4. A typical SER spectrum of R6G is presented in Figure S5. As it can be seen in the inset of Figure S5, the variation of the most intense vibrational peak of R6G (1508 cm^−1^) was less than 10%. The heat map, created using the intensities of this peak, collected on different regions of the substrate, is a clear evidence of that. A value of ~3 × 10^3^ has been calculated for substrate’s enhancement factor (EF). This value has been obtained by comparing Raman (Figure S6) and SER spectra of R6G. All the details necessary for EF calculation are presented in the Supplementary Information section.

For a proper assessment of inter-batch reproducibility capacities of our substrates we have synthesized and purified 3 batches of colloids (batch #1, batch #2 & batch #3) using the protocol described in the materials and methods section. Five different solid substrates have been prepared using each batch of TFF purified colloidal silver. Then, a blood plasma sample collected from one donor, has been measured on each solid substrate in order to evaluate inter- and intra-batch reproducibility of SER spectra. In order to prove the spectral reproducibility, we have plotted the bands intensity in counts/(mW × s) units. The linear dependence of SERS intensity with the laser power (measured at the sample surface) in the case of the most intense vibrational band of plasma (640 cm^−1^) is shown in Figure S7. In Figure S8–10, we plotted the SER spectra collected on 15 substrates created using colloidal batch #1, batch #2 respectively batch#3 (5 substrates for each batch). Each spectrum represents the mean value of 25 individual spectra collected on different regions of the substrate. Prior to any measurements, we recorded the SER spectra of the bare substrates. The mean value is also included in Figures S8–S10. All vibrational peaks identified in the spectra belong to blood plasma components. The contribution of the naked substrate to the SERS signal is negligible. The main peak in the SER spectrum of the substrate (1056 cm^−1^) might be assigned to the nitrate group from the silver salt precursor. It has an intensity of some tens of times smaller, as compared to the blood plasma peaks. Moreover, no peak or shoulder at this wavenumber can be noticed in the plasma spectra. A careful examination of the recorded spectra indicates that the ratio between the intensities of the two most intense vibrational peaks (i.e., 640 & 1134 cm^−1^) has a value between 1.36 and 1.51 (~5% of variation around the mean value of 1.43).

Inter-batch reproducibility was evaluated by plotting the mean values of the SER spectra collected on the substrates created using batch #1, batch #2 & batch #3 (Figure S11). All the spectra have been recorded on the same blood plasma sample collected from one donor. As it can be seen in the figure, the two spectra are almost identical. This superposition is a clear evidence of inter-batch reproducibility capacities of the substrates, especially in the case of SERS measurements performed on biofluids. This high degree of reproducibility represents a necessary condition that needs to be fulfilled prior to any “diagnostic relevant” PCA-LDA analysis.

**Figure 3:**
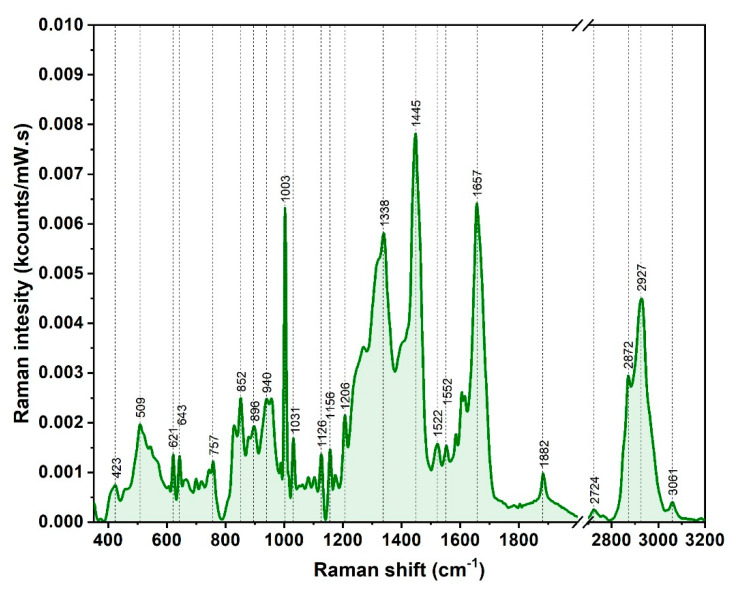
Typical Raman spectrum of blood plasma acquired using a 785 nm excitation wavelength.

A typical Raman spectrum of one plasma sample is presented in Figure 3. The spectrum has been recorded in out of resonance conditions, using an excitation wavelength of 785 nm (NIR domain), capable to reduce the inherent fluorescence background, in the 350–3200 cm^−1^ spectral interval. As expected from previous reported Raman/SERS studies perform on blood samples the spectrum is dominated by large proteins, the best confirmation being the presence of the two protein specific bands located at 1650 cm^−1^ (Amide I) and 1250 cm^−1^ (Amide III) [15]. The similarity of the Raman/SERS spectra of the plasma samples collected from healthy donors and cancer patients is one of the major scientific problem that needs to be addressed in order to allow the use of Raman/SERS-based techniques in clinical practice. We deeply believe that the answer to this stringent question can be found by designing a suitable plasmonic substrate for SERS measurements on biofluids.

The most intense band in the Raman spectrum (1445 cm^−1^) can be assigned to CH_2_/CH_3_ deformations together with the three bands located in the 2800–3100 cm^−1^ region. These bands were also detected in the case of a liposomal suspension containing a lipidic bilayer [29,30] and they suggest the presence of different types of lipids in plasma. The second most intense peak in the Raman spectrum can be assigned to an aromatic amino acid (Phenylalanine ~1003 cm^−1^), commonly present in the spectrum of all types of biofluids. These findings are very similar with those presented in the scientific literature [8,9,10,11,12,13,14,15,16] and point out the fact that Raman spectra of plasma samples do not contain specific and diagnostic relevant information.

In order to enhance the spectral information that could be provided by small biomolecules, SERS measurements have been performed on plasma samples collected from healthy donors and BC patients by using the solid silver plasmonic substrates (SSPS) synthesized in this study.

**Figure 4:**
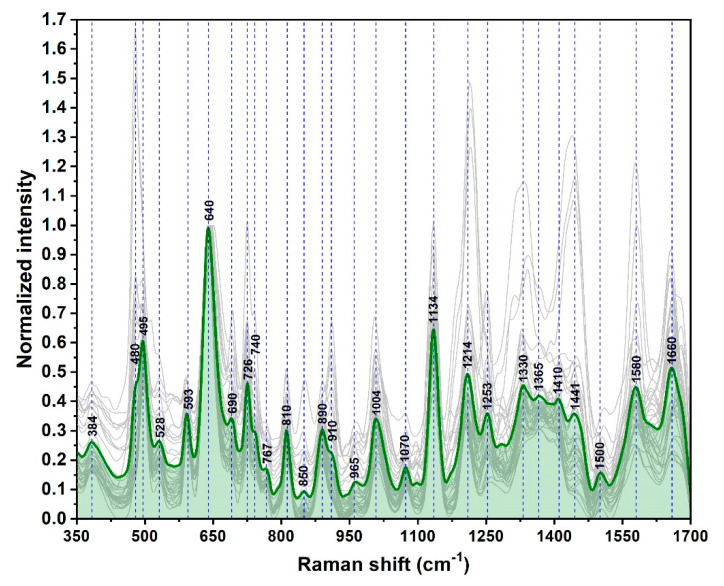
Surface Enhanced Raman (SER) spectra of blood plasma samples obtained from healthy donors (*n* = 35) recorded on the solid silver plasmonic substrates (SSPSs) using a 785 nm excitation laser. The mean spectrum is highlighted in green. The spectra were normalized to the 640 cm^−1^ peak.

In Figure 4, the SER spectra of 35 blood plasma samples collected from healthy donors are presented. In the light of previous studies, careful examination leads to the idea that xanthine derivatives have a major contribution to the vibrational spectra of these samples. As such, the 640, 725, and 1248 cm^−1^ peaks may be attributed to ring stretching vibrations of uric acid and hypoxanthine whilst 495, 811, 890, 1132, and 1205 cm^−1^ peaks may be attributed to uric acid. The 954 & 1331 cm^−1^ peaks can be assigned to hypoxanthine [15,16]. The amino acid fraction, especially aromatic amino acids such as tyrosine and phenylalanine, also have an important contribution to SER spectra. These two amino acids could be responsible for 640 and 725 cm^−1^ twisting vibrational peaks. They might have individual contributions to 848, and 1132 cm^−1^ (tyrosine) and 1004, 1205, and 1575 cm^−1^ (phenylalanine). On the other hand, purine bases (adenine, guanine) bring a smaller but still relevant contribution to the SER spectral pattern of blood plasma samples. The stretching ring vibration of guanine contributes to the 495 cm^−1^ peak, whereas the bending C–H vibrations of adenine molecules contribute to 725 and 1332 cm^−1^ peaks [31,32,33,34,35,36,37].

Other small molecules, belonging to a multitude of biochemical classes such as amino acids (proline, L-serine, tryptophan), oligopeptides (glutathione), saccharides (D-mannose, D-galactosamine), lipids, vitamins (riboflavin, ascorbic acid) can contribute to the SER spectral pattern of blood plasma, as it has been previously suggested [30,31,32,33,34,35].

Given the aforementioned arguments one can conclude that the main contributions in the SER spectra of blood plasma samples collected from healthy donors and recorded on our SSPSs are due to small non-protein biomolecules (molecular mass below 1 kDa). A possible explanation for this finding could be their higher affinity for this type of nanostructured solid substrate.

**Figure 5:**
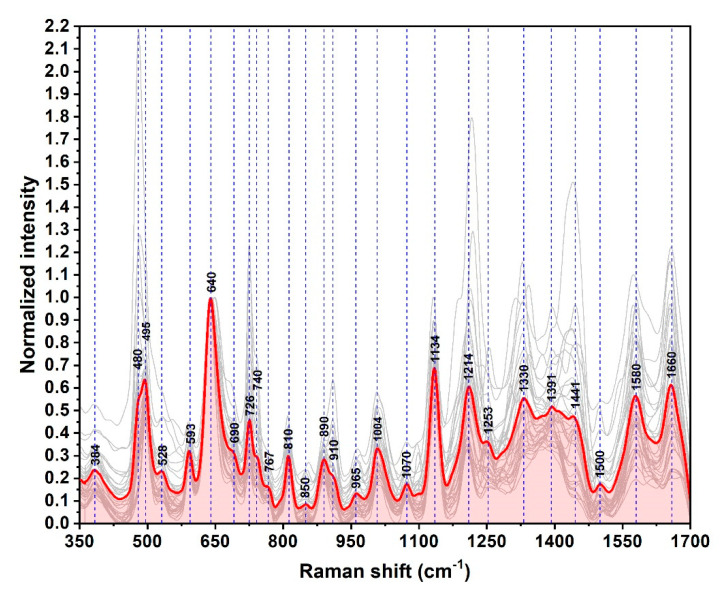
SER spectra of blood plasma samples obtained from BC patients (*n* = 29) recorded on SSPSs using a 785 nm excitation laser. The spectra were normalized to the 640 cm^−1^ peak.

**Figure 6:**
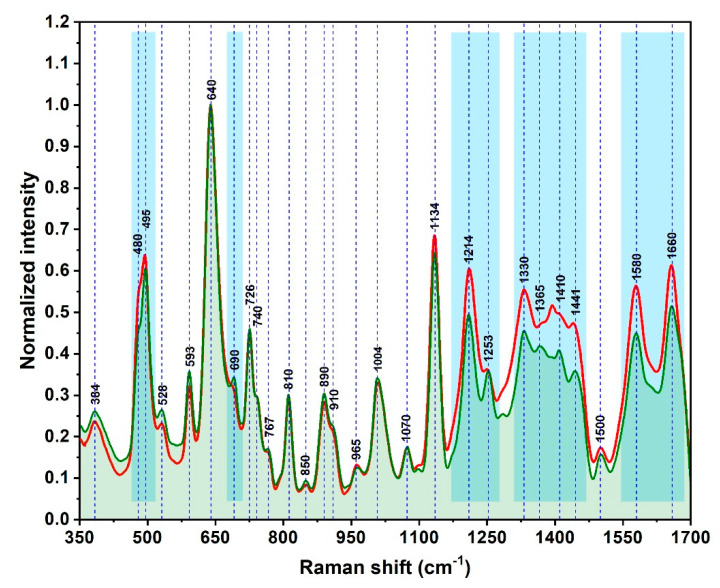
Overlapping of the mean SER spectra recorded on the blood plasma samples collected from healthy subjects (green spectrum) and BC patients (red spectrum) recorded on SSPSs using a 785 nm excitation laser. The spectra were normalized to the 640 cm^−1^ peak.

The same phenomena were detected in the case of the plasma samples collected from 29 BC patients (Figure 5). The spectra have been recorded in the same conditions and on the same type of substrates. The vast majority of the vibrational bands can be found in both spectra by superposing the normalized mean spectra recorded on donors and BC patients’ samples (Figure 6). The small intensity differences can be detected in the blue highlighted zones from Figure 6. These small spectral differences are unable to clearly differentiate the BC patients from the healthy donors, but the quality and high degree of reproducibility of the SER spectra represent a very good starting point for the Multivariate Analysis.

**Figure 7:**
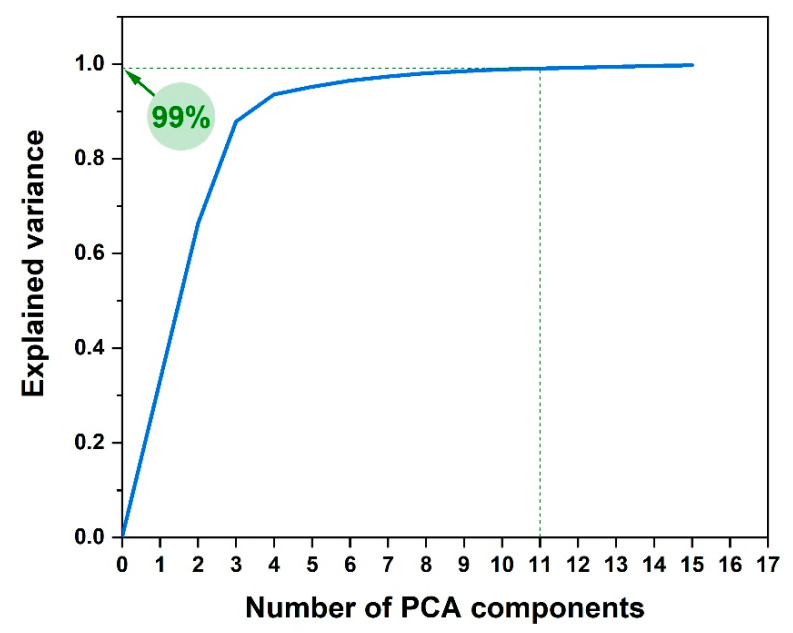
Explained variance as a function of Principal Component Analysis (PCA) components.

Therefore, a combined Principal Component Analysis-Linear Discriminant Analysis (PCA-LDA) of all SER spectra was performed in order to test the capacity of this multivariate approach to separate the two groups of samples: BC versus control. PCA is used to transform the data into a lower-dimensional space such that the explained variance is maximized for the new dimensionality. This is achieved by selecting a number of components—perpendicular linear directions in the original space—and projecting the data onto them. By choosing these components to maximize the variance retained in the new space, PCA essentially filters out a large number of noise dimensions. Figure 7 shows the relationship between the percentage of explained variance and the number of PCA components (i.e., dimensionality of the new space). In our experiments, we chose 11 components which explain 99.09% of the variance. In Figure S12 we plotted the loading curves for the first 15 components. As can be seen, the loading curve for the 11th component shows identifiable spectral features besides the noise. As a result, choosing 11 components does not lead to any overfitting.

The spectral data, transformed by PCA, was used as input for an LDA discriminator, which finds a linear boundary between BC and control. This boundary is obtained by maximizing the ratio between the between-class variance and within-class variance.

The dataset consisted of 64 data points represented by 35 control and 29 BC patients. Because of the relatively small dataset, Leave-One-Out Cross-Validation (LOOCV) was used to perform the analysis, i.e., the PCA and LDA were trained on all but one data point; the left-out data point was then processed by PCA and had its label (cancer/control) predicted by LDA. The true and predicted classes were recorded for each individual data point using this scheme. The results of PCA-LDA analysis, obtained by LOOCV, are presented in Table 1.

As it can be seen in Table 1, the BC patients can be distinguished from healthy donors with a sensitivity (S) of 90%, a specificity (S) of 89% and an accuracy (A) of 89%, The results are very promising given that mammography, which represents today the golden standard in BC detection, can only detect BC with a sensitivity of 70% and a specificity of 75% [34]. The reported sensitivity, specificity and accuracy values are very close to those reported by Cervo et al. [15]. In our case, the experimental spectra have been recorded on pristine plasma samples whereas in their case filtered serum samples have been used. By removing this additional step of blood sample filtration, the here proposed discrimination method has the potential of easier implementation in clinical applications for BC or different types of cancer but further studies are still needed.

## 4. Conclusions

In this paper, we propose a very simple, cheap and efficient method for the synthesis of solid plasmonic substrates. The use of Leopold-Lendl Ag colloids purified and concentrated by means of the TFF method, drop-casted in controlled temperature conditions on a silent Raman CaF_2_ glass. To the best of our knowledge this is the first time, when such a substrate is proposed in the literature. Using these very simple and cheap conditions, we obtained highly reproducible SERS signals for a very complex analyte like blood plasma.

Once synthesized, the reproducibility of the spectra generated by the substrate were tested on a typical Raman analyte as well as on a blood plasma sample. Then the substrates were used for recording SER spectra from pristine blood plasma samples collected from 35 healthy donors and 29 BC patients. A PCA-LDA analysis was used to statistically differentiate the SER spectra, collected from the two clinically different groups. This allowed the distinction between healthy donors and BC patients with high sensitivity and specificity (90% sensitivity and 89% specificity). Without performing any other purification or deproteinization step, a diagnostic accuracy of 89% was obtained performing the SERS measurements directly on blood plasma samples. This a direct consequence of SSPSs ability to generate reproducible and diagnostic relevant spectral information.

In conclusion, the solution to the reproducibility problem of SERS analysis can be resolved by the substrate and its subsequent optimization. In the future, we hope that our results generate enough interest that this type of solid silver substrates could also be employed for SERS based detection of other types of cancer.

## Figures and Tables

**Table 1:** Results of Principal Component Analysis-Linear Discriminant Analysis (PCS-LDA) analysis performed on all SER spectra.

Accuracy	Precision	Sensitivity	Specificity	True Positives	True Negatives	False Positives	False Negatives
0.89	0.87	0.90	0.89	26	31	4	3

## Supplementary Materials

The following are available online at https://www.mdpi.com/2079-4991/10/6/1212/s1, Figure S1: TEM image and size distribution plot of the purified quasi-spherical AgHya NPs, Figure S2: EDS analysis of purified silver colloids, Figure S3: A typical SERS spectrum of methylene blue (MB) 10^−5^ M recorded, using a 785 nm excitation laser, Figure S4: Optical image of three different areas of the plasmonic substrate covered with R6G analyte, Figure S5: SER spectrum of rhodamine 6G (R6G) 10^−3^ M recorded using an excitation laser of 785 nm. The upper insets present a heat map, showing a very small variation of the most intense vibrational peak of R6G (1508 cm^−1^). The lower inset presents an optical image of the substrate where the spectra were recorded, Figure S6: Raman spectrum of rhodamine 6G (R6G) 10^−3^ M recorded using a 785 nm excitation laser, Figure S7: Variation of the most intense vibrational band of blood plasma (640 cm^−1^) as a function of excitation laser intensity measured at the sample surface, Figure S8: SER spectra of blood plasma samples collected on five different solid substrates created using the same purified colloidal solution (batch 1). Each spectrum represents the mean of 25 individual spectra recorded on different regions of the substrate. The mean SER spectrum recorded on the 5 bare substrates is also plotted for comparison. All the spectra were recorded using the same experimental conditions. The spectra were offset for clarity, Figure S9: SER spectra of blood plasma samples collected on five different solid substrates created using the same purified colloidal solution (batch 2). Each spectrum represents the mean of 25 individual spectra recorded on different regions of the substrate. The mean SER spectrum of the five bare substrates is also plotted for comparison. All the spectra were recorded using the same experimental conditions. The spectra were offset for clarity, Figure S10: SER spectra of blood plasma samples collected on five different solid substrates created using the same purified colloidal solution (batch 3). Each spectrum represents the mean of 25 individual spectra recorded on different regions of the substrate. The mean SER spectrum of the five bare substrates is also plotted for comparison. All the spectra were recorded using the same experimental conditions. The spectra were offset for clarity, Figure S11. Superposition of the mean spectra collected on the substrates fabricated using the colloidal batch #1 (red spectrum), #2 (blue spectrum) and #3 (green spectrum). All the spectra were recorded using the same experimental conditions on the same plasma sample, Figure S11. Loading curves of the first 15 principal components.

## References

[1] Weller D., Vedsted P., Rubin G., Walter F.M., Emery J., Scott S., Campbell C., Andersen R.S., Hamilton W., Olesen F. (2012). The Aarhus statement: Improving design and reporting of studies on early cancer diagnosis. Br. J. Cancer.

[2] Tirpe A.A., Gulei D., Ciortea S.M., Crivii C., Berindan-Neagoe I. (2019). Hypoxia: Overview on hypoxia-mediated mechanisms with a focus on the role of hif genes. Int. J. Mol. Sci..

[3] Gøtzsche P.C., Olsen O. (2000). Is screening for breast cancer with mammography justifiable?. Lancet.

[4] Olsen O., Gøtzsche P.C. (2001). Cochrane review on screening for breast cancer with mammography. Lancet.

[5] Bleyer A., Welch H.G. (2012). Effect of three decades of screening mammography on breast-cancer incidence. N. Engl. J. Med..

[6] Vogel V.G. (2018). Epidemiology of Breast Cancer. The Breast.

[7] Moisoiu V., Stefancu A., Gulei D., Boitor R., Magdo L., Raduly L., Pasca S., Kubelac P., Mehterov N., Chiș V. (2019). SERS-based differential diagnosis between multiple solid malignancies: Breast, colorectal, lung, ovarian and oral cancer. Int. J. Nanomed..

[8] Premasiri W.R., Lee J.C., Ziegler L.D. (2012). Surface-enhanced Raman scattering of whole human blood, blood plasma, and red blood cells: Cellular processes and bioanalytical sensing. J. Phys. Chem. B..

[9] Harris A.T., Lungari A., Needham C.J., Smith S.L., Lones M.A., Fisher S.E., Yang X.B., Cooper N., Kirkham J., Smith D.A. (2009). Potential for Raman spectroscopy to provide cancer screening using a peripheral blood sample. Head Neck Oncol. Bio. Med. Central..

[10] Baker M.J., Hussain S.R., Lovergne L., Untereiner V., Hughes C., Lukaszewski R.A., Thiéfin G., Sockalingum G.D. (2016). Developing and understanding biofluid vibrational spectroscopy: A critical review. Chem. Soc. Rev..

[11] Butler H.J., Ashton L., Bird B., Cinque G., Curtis K., Dorney J., Esmonde-White K., Fullwood N.J., Gardner B., Martin-Hirsch P.L. (2016). Using Raman spectroscopy to characterize biological materials. Nat. Protoc..

[12] Baker M.J., Byrne H.J., Chalmers J., Gardner P., Goodacre R., Henderson A., Kazarian S.G., Martin F.L., Moger J., Stone N. (2018). Clinical applications of infrared and Raman spectroscopy: State of play and future challenges. Analyst.

[13] Bonifacio A., Cervo S., Sergo V. (2015). Label-free surface-enhanced Raman spectroscopy of biofluids: Fundamental aspects and diagnostic applications. Anal. Bioanal. Chem..

[14] Enejder A.M.K., Koo T.-W., Oh J., Hunter M., Sasic S., Feld M.S., Horowitz G.L. (2002). Blood analysis by Raman spectroscopy. Opt. Lett..

[15] Bonifacio A., Dalla Marta S., Spizzo R., Cervo S., Steffan A., Colombatti A., Sergo V. (2014). Surface-enhanced Raman spectroscopy of blood plasma and serum using Ag and Au nanoparticles: A systematic study. Anal. Bioanal. Chem..

[16] Cervo S., Mansutti E., Del Mistro G., Spizzo R., Colombatti A., Steffan A., Sergo V., Bonifacio A. (2015). SERS analysis of serum for detection of early and locally advanced breast cancer. Anal. Bioanal. Chem. Springer.

[17] Banholzer M.J., Millstone J.E., Qin L., Mirkin C.A. (2008). Rationally designed nanostructures for surface-enhanced Raman spectroscopy. Chem. Soc. Rev..

[18] Stiufiuc R., Iacovita C., Lucaciu C.M., Stiufiuc G., Dutu A.G., Braescu C., Leopold N. (2013). SERS-active silver colloids prepared by reduction of silver nitrate with short-chain polyethylene glycol. Nanoscale Res. Lett. Springer.

[19] Stiufiuc R., Toderas F., Iosin M., Stiufiuc G. (2010). Anisotropic gold nanocrystals: Synthesis and characterization. Int. J. Mod. Phys. B.

[20] Stiufiuc R., Iacovita C., Nicoara R., Stiufiuc G., Florea A., Achim M., Lucaciu C.M. (2013). One-step synthesis of PEGylated gold nanoparticles with tunable surface charge. J. Nanomater. Hindawi..

[21] Stiufiuc G., Toma V., Moldovan A., Stiufiuc R., Lucaciu M. (2017). One pot microwave assisted synthesis of cyclodextrins capped spherical gold nanoparticles. Dig. J. Nanomater. Biostructures.

[22] Nițică Ștefan Moldovan A.I., Toma V., Moldovan C.S., Berindan-Neagoe I., Știufiuc G., Lucaciu C.M., Știufiuc R. (2018). PEGylated gold nanoparticles with interesting plasmonic properties synthesized using an original, rapid, and easy-to-implement procedure. J. Nanomater. Hindawi..

[23] Stiufiuc R., Iacovita C., Stiufiuc G., Bodoki E., Chis V., Lucaciu C.M. (2015). Surface mediated chiral interactions between cyclodextrins and propranolol enantiomers: A SERS and DFT study. Phys. Chem. Chem. Phys..

[24] Bodoki E., Oltean M., Bodoki A., Ştiufiuc R. (2012). Chiral recognition and quantification of propranolol enantiomers by surface enhanced Raman scattering through supramolecular interaction with β-cyclodextrin. Talanta. Elsevier.

[25] Leopold N., Lendl B. (2003). A new method for fast preparation of highly surface-enhanced Raman scattering (SERS) active silver colloids at room temperature by reduction of silver nitrate with hydroxylamine hydrochloride. J. Phys. Chem. B..

[26] Anders C.B., Baker J.D., Stahler A.C., Williams A.J., Sisco J.N., Trefry J.C., Wooley D.P., Pavel Sizemore I.E. (2012). Tangential flow ultrafiltration: A “green” method for the size selection and concentration of colloidal silver nanoparticles. J. Vis. Exp..

[27] Dorney K.M., Baker J.D., Edwards M.L., Kanel S.R., O’Malley M., Pavel Sizemore I.E. (2014). Tangential flow filtration of colloidal silver nanoparticles: A “Green” laboratory experiment for chemistry and engineering students. J. Chem. Educ..

[28] Trefry J.C., Monahan J.L., Weaver K.M., Meyerhoefer A.J., Markopolous M.M., Arnold Z.S., Wooley D.P., Pavel I.E. (2010). Size selection and concentration of silver nanoparticles by tangential flow ultrafiltration for SERS-based biosensors. J. Am. Chem. Soc..

[29] Stiufiuc R., Iacovita C., Stiufiuc G., Florea A., Achim M., Lucaciu C.M. (2015). A new class of pegylated plasmonic liposomes: Synthesis and characterization. J. Colloid Interface Sci..

[30] Știufiuc G.F., Nițică S., Toma V., Iacoviță C., Zahn D.R.T., Tetean R., Burzo E., Lucaciu C.M., Știufiuc R.I. (2019). Synergistical use of electrostatic and hydrophobic interactions for the synthesis of a new class of multifunctional nanohybrids: Plasmonic magneto-liposomes. Nanomaterials.

[31] Lin D., Pan J., Huang H., Chen G., Qiu S., Shi H., Chen W., Yu Y., Feng S., Chen R. (2014). Label-free blood plasma test based on surface-enhanced Raman scattering for tumor stages detection in nasopharyngeal cancer. Sci. Rep..

[32] Feng S., Pan J., Wu Y., Lin D., Chen Y., Xi G., Lin J., Chen R. (2011). Study on gastric cancer blood plasma based on surface-enhanced Raman spectroscopy combined with multivariate analysis. Sci. China Life Sci..

[33] Lin J., Chen R., Feng S., Pan J., Li Y., Chen G., Cheng M., Huang Z., Yu Y., Zeng H. (2011). A novel blood plasma analysis technique combining membrane electrophoresis with silver nanoparticle-based SERS spectroscopy for potential applications in noninvasive cancer detection. Nanomedicine.

[34] González-Solís J., Luévano-Colmenero G., Vargas-Mancilla J. (2013). Surface enhanced Raman spectroscopy in breast cancer cells. Laser.

[35] Feng S., Lin D., Lin J., Li B., Huang Z., Chen G., Zhang W., Wang L., Pan J., Chen R. (2013). Blood plasma surface-enhanced Raman spectroscopy for non-invasive optical detection of cervical cancer. Analyst.

[36] Li S., Li L., Zeng Q., Zhang Y., Guo Z., Liu Z., Jin M., Su C., Lin L., Xu J. (2015). Characterization and noninvasive diagnosis of bladder cancer with serum surface enhanced Raman spectroscopy and genetic algorithms. Sci. Rep..

[37] Han X.X., Ozaki Y., Zhao B. (2012). Label-free detection in biological applications of surface-enhanced Raman scattering. TrAC Trends Anal. Chem. Elsevier.

