# Supplementary material for: Solid Plasmonic Substrates for Breast Cancer Detection by Means of SERS Analysis of Blood Plasma
Source: Nanomaterials (Basel). 2020 Jun 21;10(6):1212. doi: 10.3390/nano10061212 (PMC7353077; doi:10.3390/nano10061212)
Supplement: Supplementary file 1 [file nanomaterials-10-01212-s001.docx]

Supplementary Materials

Solid Plasmonic Substrates for Breast Cancer Detection by Means of SERS Analysis of Blood Plasma

Gabriela Fabiola Știufiuc ^1,ϯ^, Valentin Toma ^2,ϯ^, Mihail Buse ^2^, Radu Mărginean ^2^, Gabriela Morar-Bolba ^3^, Bogdan Culic ^4^, Romulus Tetean ^1^, Nicolae Leopold ^1^, Ioana Pavel ^5^, Constantin Mihai Lucaciu ^6,^* and Rareș Ionuț Știufiuc ^2,6,^*

^1^ Faculty of Physics, “Babeș-Bolyai” University, M. Kogălniceanu 1, 400084 Cluj-Napoca, Romania; gabi.stiufiuc@phys.ubbcluj.ro (G.F.Ș.); romulus.tetean@phys.ubbcluj.ro (R.T.); nicolae.leopold@phys.ubbcluj.ro (N.L.)

^2^ MedFuture Research Center for Advance Medicine, “Iuliu Hațieganu” University of Medicine and Pharmacy, L. Pasteur 4-6, 400349 Cluj-Napoca, Romania; valentin.toma@umfcluj.ro (V.T.); buse.mihail@umfcluj.ro (M.B.)

^3^ Department of Senology, The Oncology Institute “Prof. Dr. Ion Chiricuta”, 34-36 Republicii Street, 400015 Cluj-Napoca, Romania; gabrielambolba@yahoo.co.uk

^4^ Department of Dental Propedeutics and Esthetics, “Iuliu Hațieganu” University of Medicine and Pharmacy, 8 V. Babes Street, 400012 Cluj-Napoca, Romania; bculic@umfcluj.ro

^5^ Department of Chemistry, Wright State University, 3640 Colonel Glenn Hwy., Dayton, OH 45435-0001, USA; ioana.pavel@wright.edu

^6^ Department of Pharmaceutical Physics-Biophysics, “Iuliu Hațieganu” University of Medicine and Pharmacy, L. Pasteur 6, 400349 Cluj-Napoca, Romania

* corresponding authors: clucaciu@umfcluj.ro (C.M.L.); rares.stiufiuc@umfcluj.ro (R.I.Ș.); Tel.: +40-74-4647-854 (C.M.L.); +40-72-6340-278 (R.S.)

^ϯ^ these authors equally contributed to this paper

Received: 11 May 2020; Accepted: date; Published: date


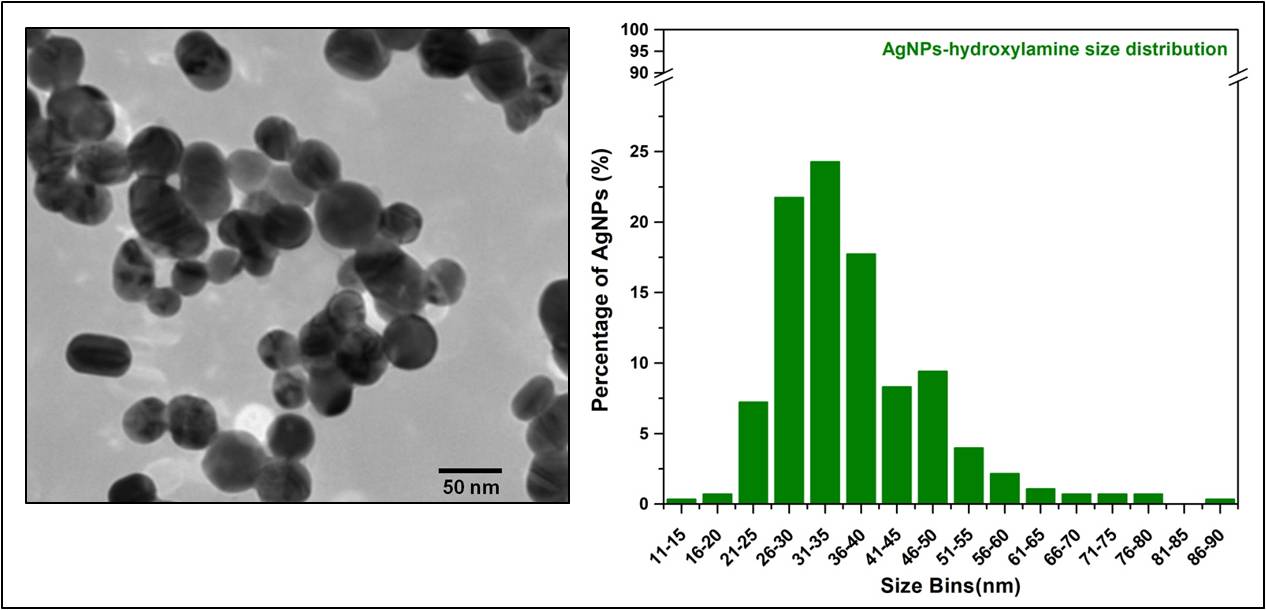


**Figure S1.** TEM image and size distribution plot of the purified quasi-spherical AgHya NPs.


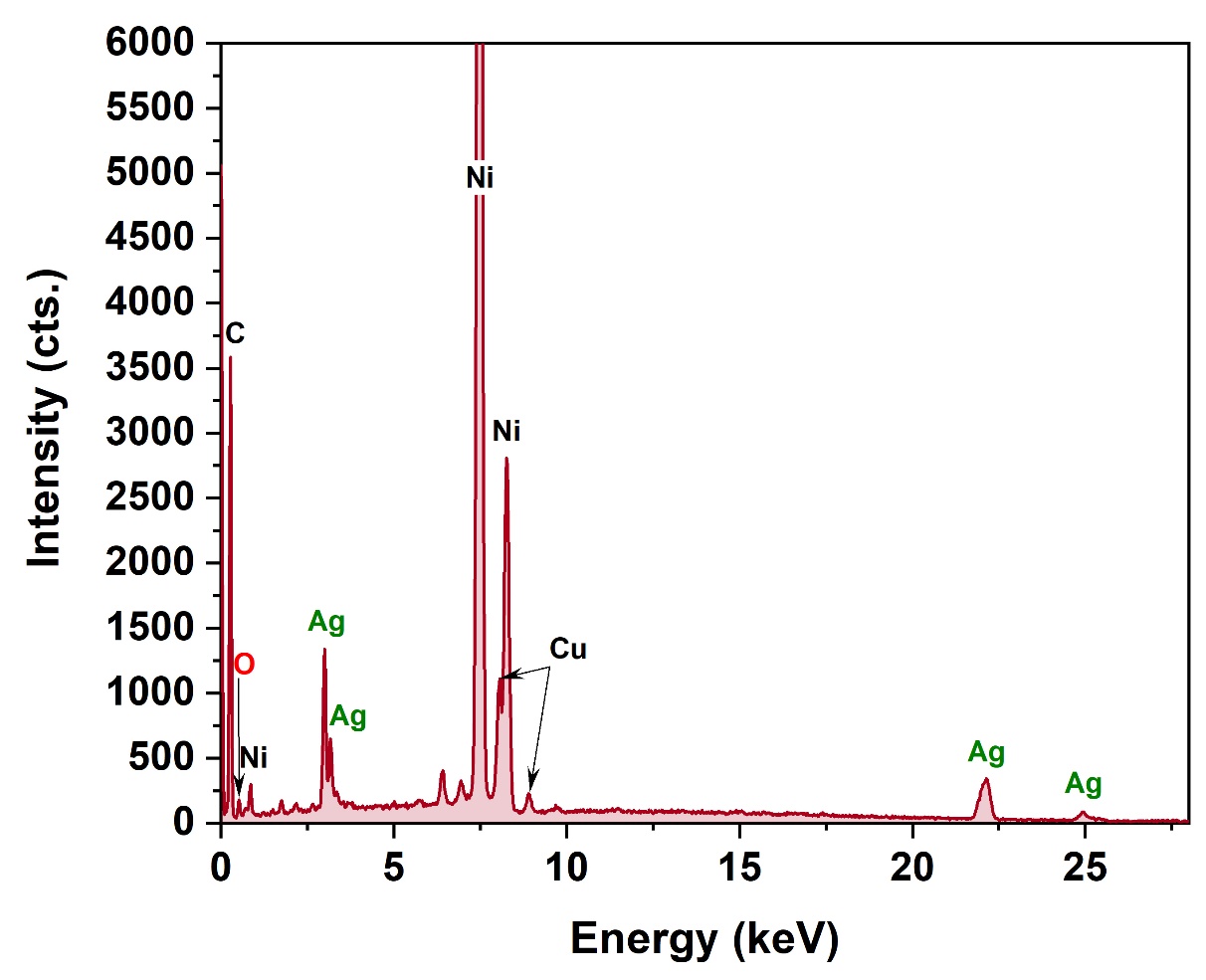


**Figure S2.** EDS analysis of purified silver colloids.


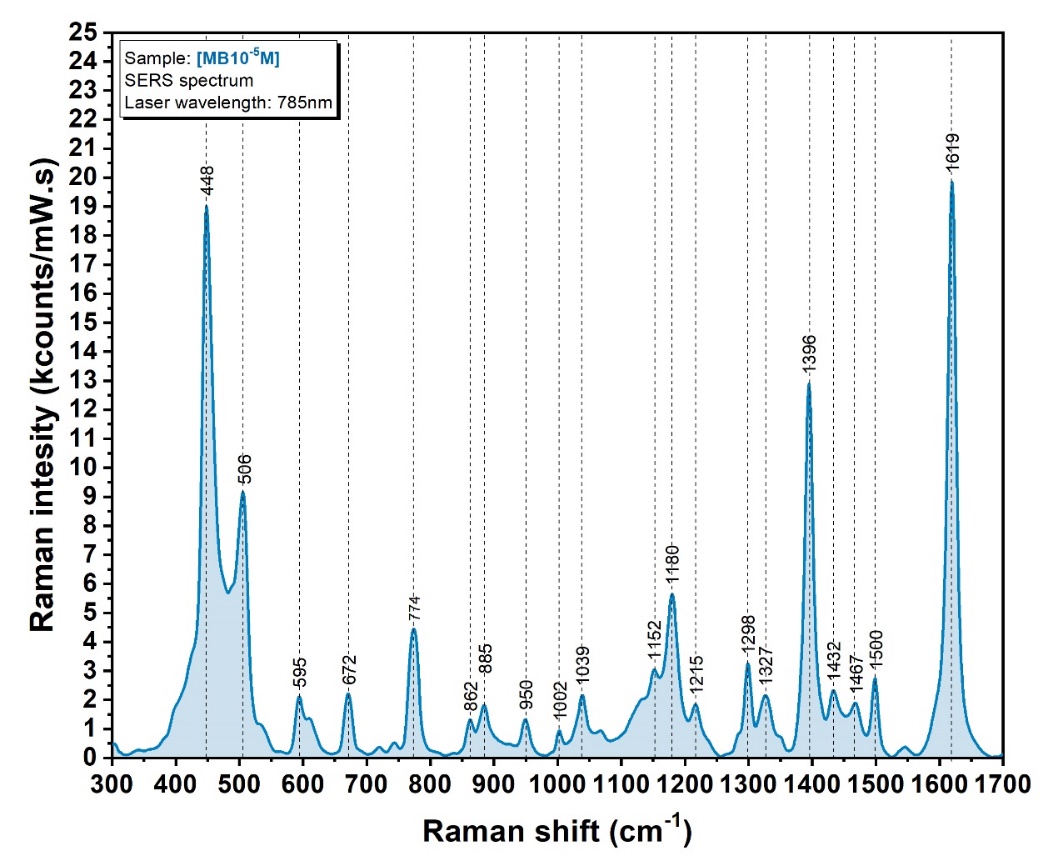


**Figure S3.** A typical SERS spectrum of methylene blue (MB) 10^−5^ M recorded using a 785 nm excitation laser.


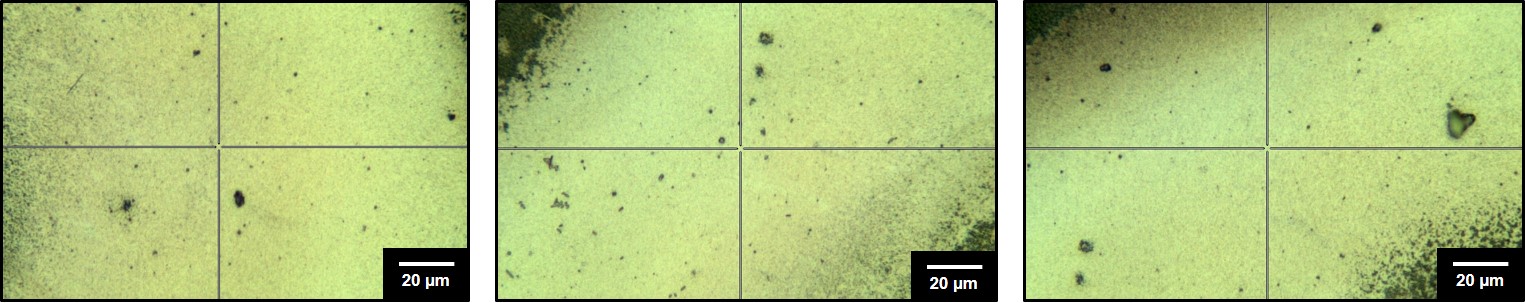


**Figure S4.** Optical image of three different areas of the plasmonic substrate covered with R6G analyte.


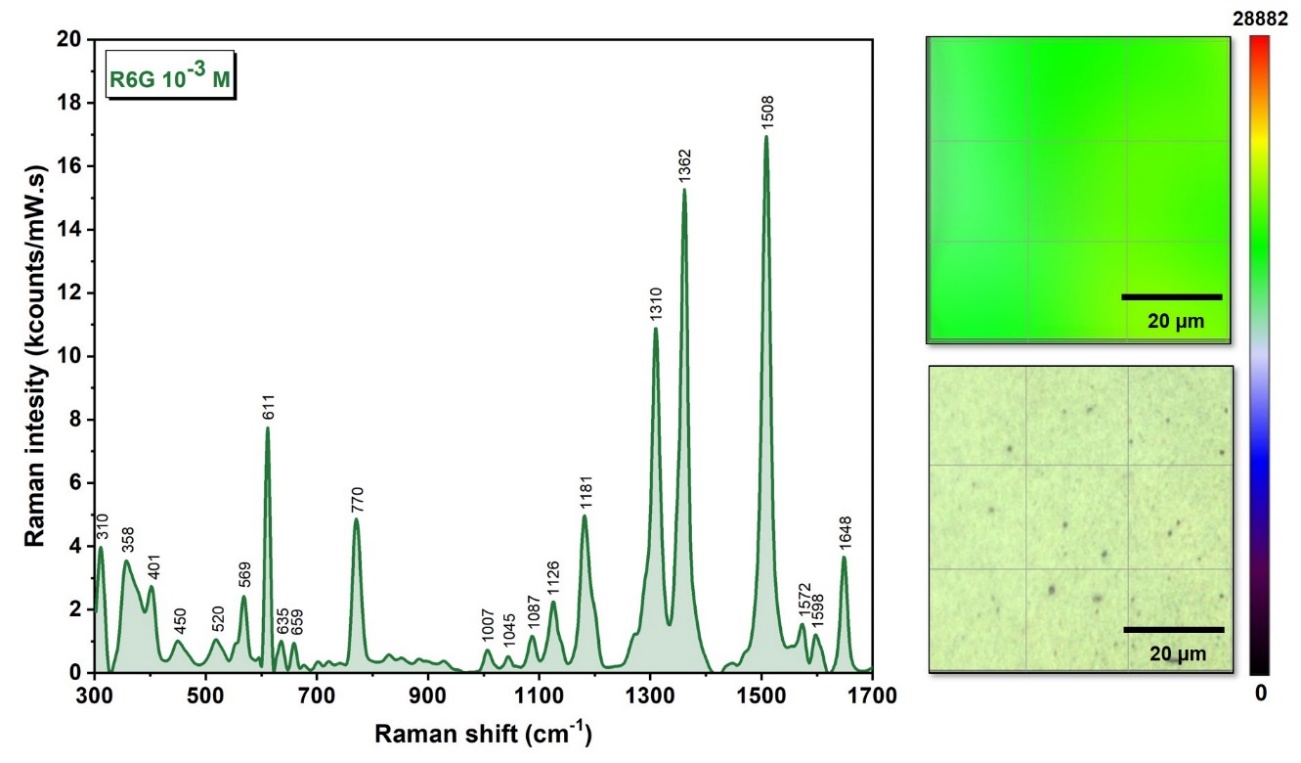


**Figure S5.** SER spectrum of rhodamine 6G (R6G) 10^−3^ M recorded using an excitation laser of 785 nm. The upper insets present a heat map, showing a very small variation of the most intense vibrational peak of R6G (1508 cm^−1^). The lower inset presents an optical image of the substrate where the spectra were recorded.


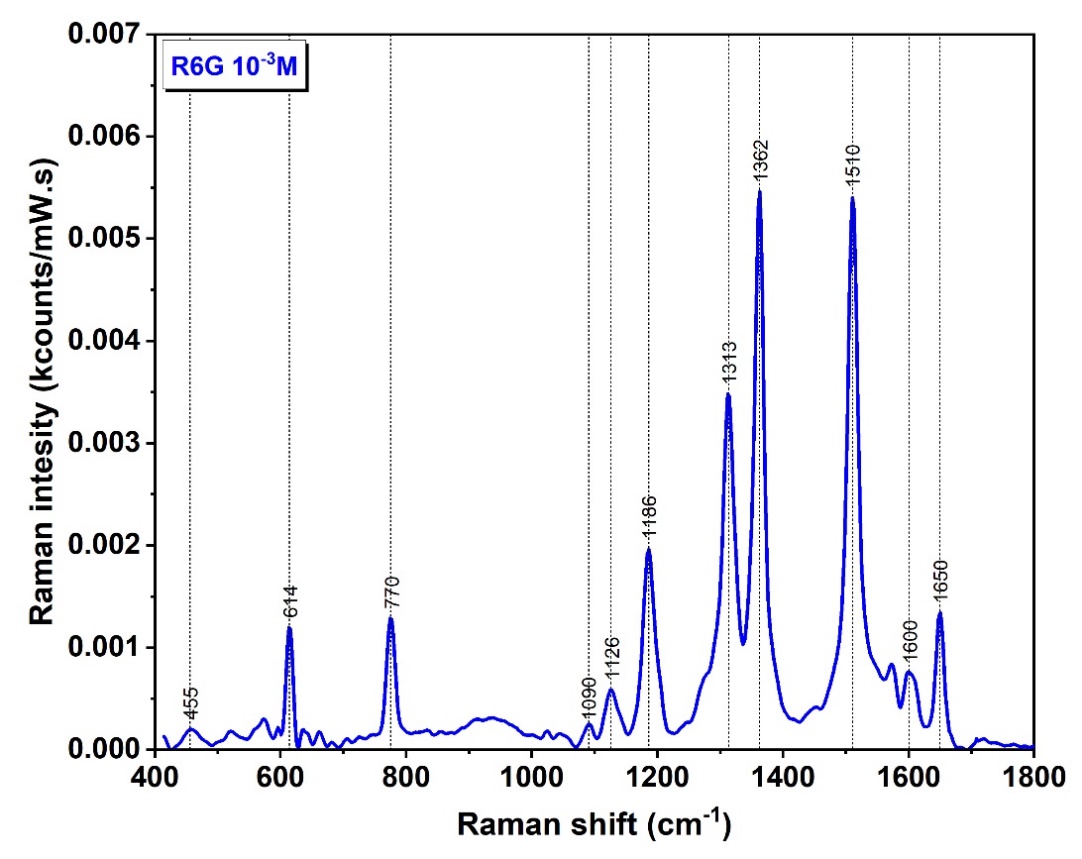


**Figure S6.** Raman spectrum of rhodamine 6G (R6G) 10^−3^ M recorded using a 785 nm excitation laser.


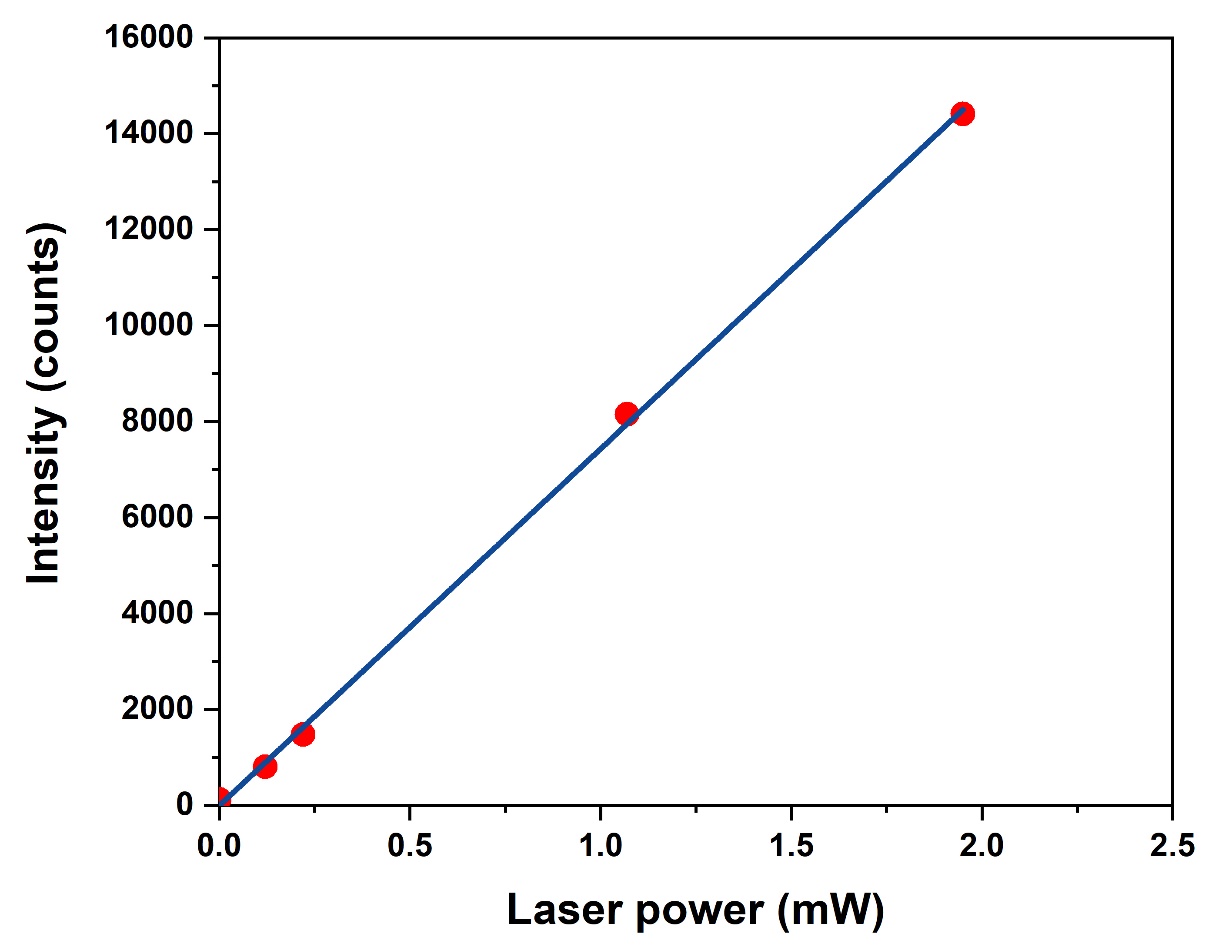


**Figure S7.** Variation of the most intense vibrational band of blood plasma (640 cm^−1^) as a function of excitation laser intensity measured at the sample surface.


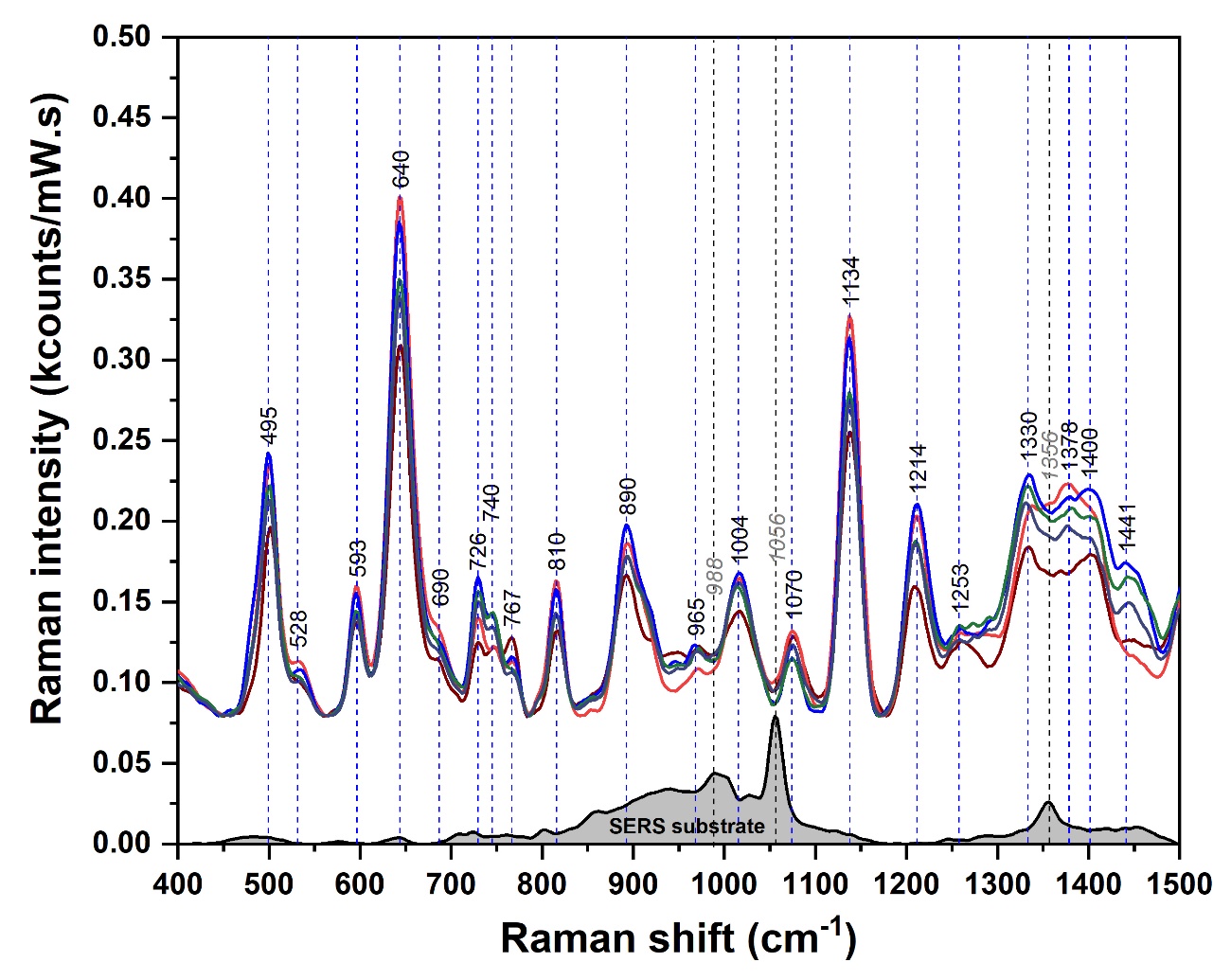


**Figure S8.** SER spectra of blood plasma samples collected on 5 different solid substrates created using the same purified colloidal solution (batch 1). Each spectrum represents the mean of 25 individual spectra recorded on different regions of the substrate. The mean SER spectrum recorded on the 5 bare substrates is also plotted for comparison. All the spectra were recorded using the same experimental conditions. The spectra were offset for clarity.


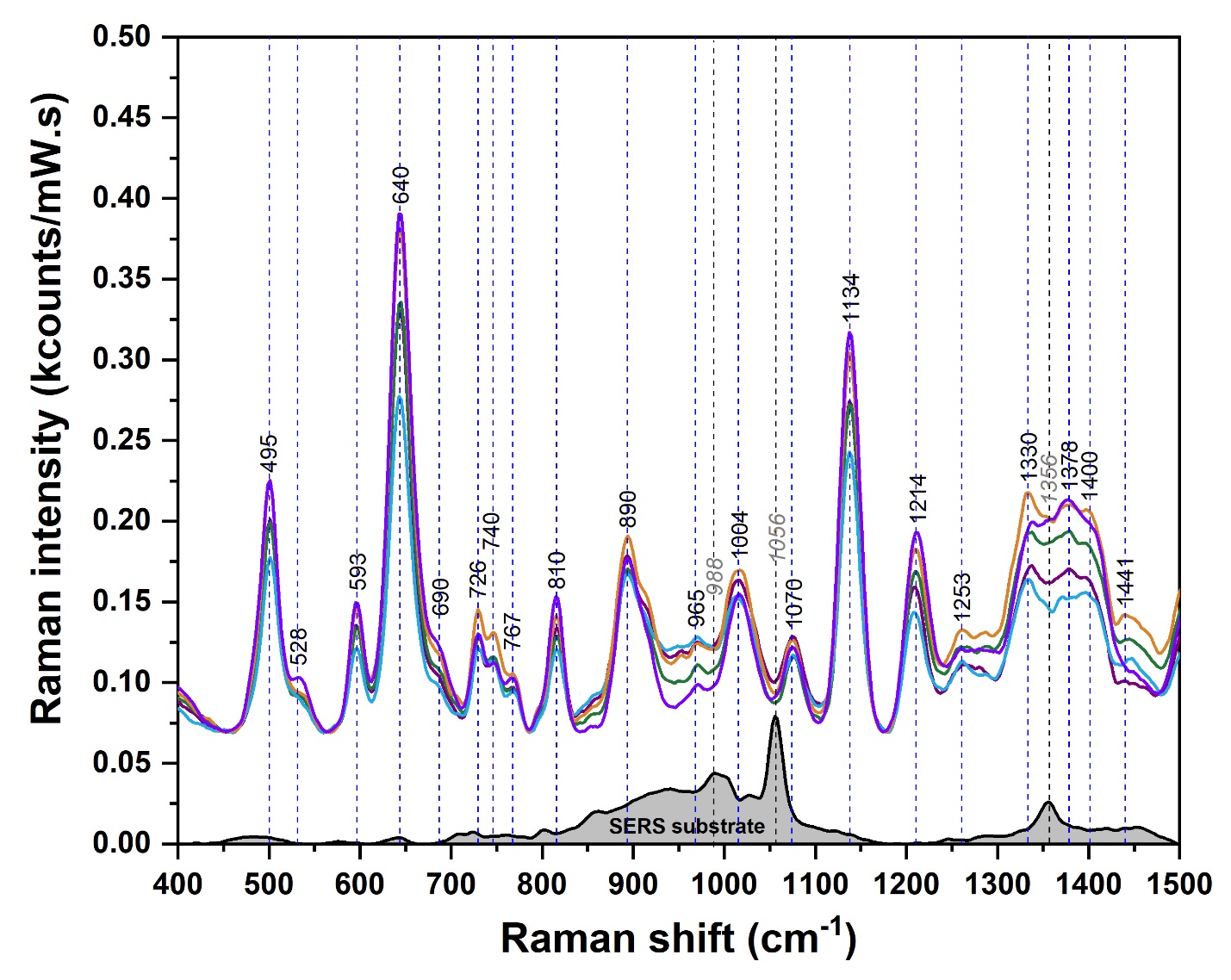


**Figure S9.** SER spectra of blood plasma samples collected on 5 different solid substrates created using the same purified colloidal solution (batch 2). Each spectrum represents the mean of 25 individual spectra recorded on different regions of the substrate. The mean SER spectrum of the 5 bare substrates is also plotted for comparison. All the spectra were recorded using the same experimental conditions. The spectra were offset for clarity.


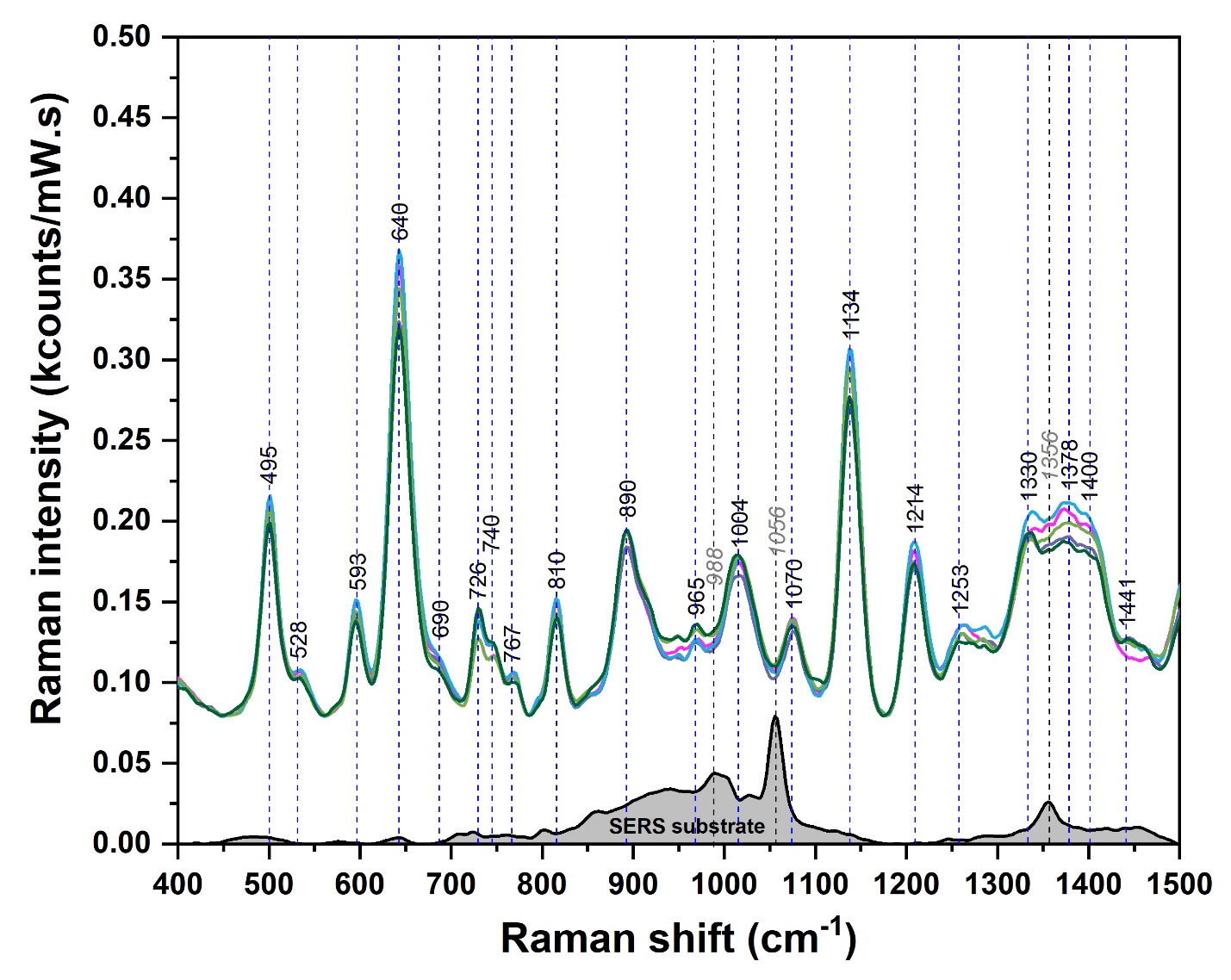


**Figure S10.** SER spectra of blood plasma samples collected on 5 different solid substrates created using the same purified colloidal solution (batch 3). Each spectrum represents the mean of 25 individual spectra recorded on different regions of the substrate. The mean SER spectrum of the 5 bare substrates is also plotted for comparison. All the spectra were recorded using the same experimental conditions. The spectra were offset for clarity.


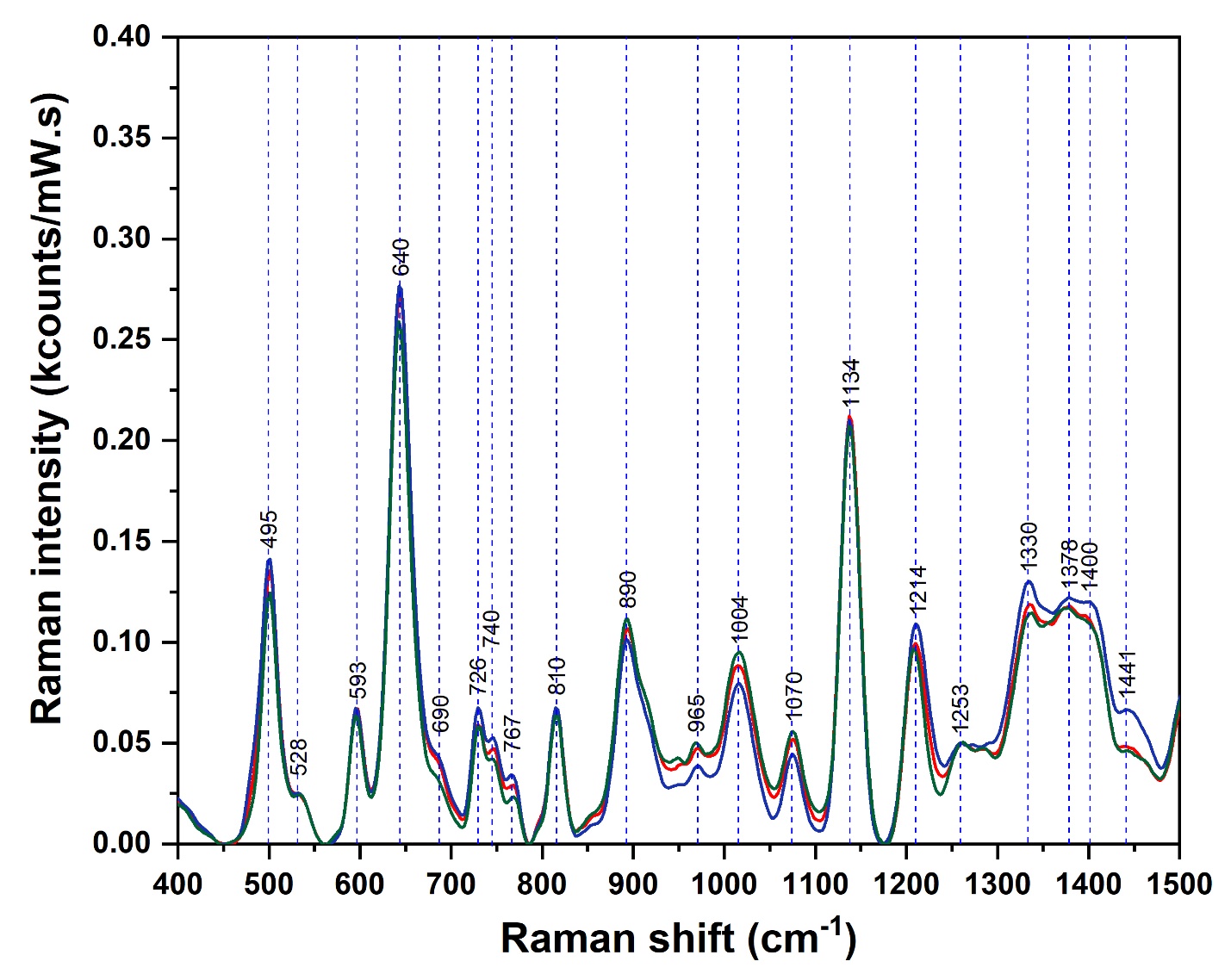


**Figure S11.** Superposition of the mean spectra collected on the substrates fabricated using the colloidal batch #1 (red spectrum) #2 (blue spectrum) and #3 (green spectrum). All the spectra were recorded using the same experimental conditions on the same plasma sample.


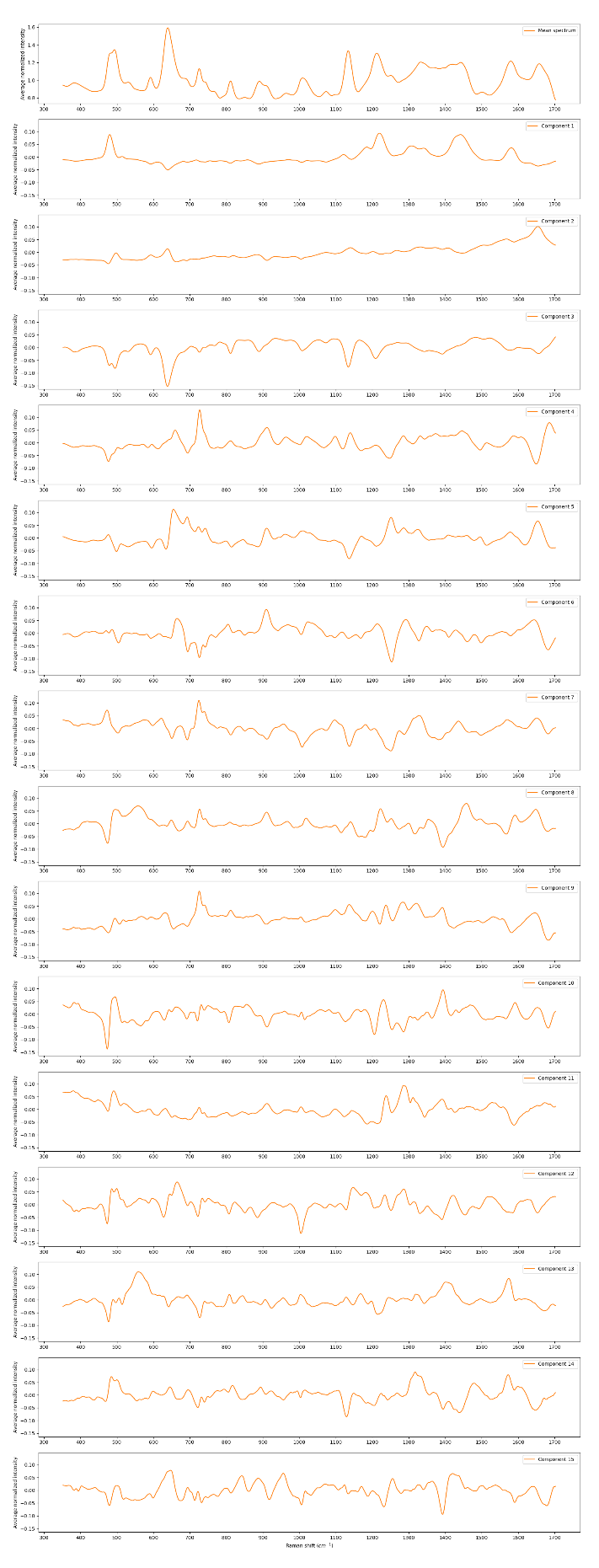


**Figure S12.** Loading curves of the first 15 principal components.

1. Calculation of the EF

In our paper we have used rhodamine 6G (R6G) molecules for the calculation of substrates’ enhancement factor (EF). According to a procedure proposed by Gupta and Weimar [1] the EF can be calculated by using the following equation:

| $EF=\frac{{M_{Raman}\times S_{Surf}\times I}_{Surf}}{{M_{Surf}\times S_{Raman}\times I}_{Raman}}$ | (1) |
| --- | --- |

where *M_Surf_* and *M_Raman_* are the numbers of molecules dropped onto the solid plasmonic substrate (SERS measurements) and CaF_2_ (Raman measurements), *S_Surface_* and *S_Raman_* are the geometrical areas of the molecular films and *I_Surf_* and *I_Raman_* are the SERS/Raman intensities of the most intense vibrational band that has been used for the calculation of EF (1508/1510 cm^−1^). Both measurements were performed using a 50× objective and an excitation laser of 785 nm. In the case of Raman measurements, a 100% laser power was used, the acquisition time was 10 s and a number of 4 acquisition was recorded. For SERS measurements the laser power was set to 0.1%, all other conditions being identical. In order to improve the accuracy of EF calculation we measured the laser intensity on the sample surface in the two cases and the nominal values were 113 mW (100 % laser power) respectively 0.22 mW (0.1% laser power). The intensities of the 1508/1510 cm^−1^ vibrational band, in both SERS and Raman spectra have been plotted in *kcounts/(*mW × s*)* units. For both measurements we have used 10^-3^ M aqueous solutions of R6G. The circular spots had a diameter of ~2 mm in both cases. The values of 1508/1510 cm^-1^ intensities were 16.9 respectively 0.0054 kcounts/(mW × s).

Using these data, the EF of the here proposed solid substrates has a value of ~3 × 10^3^.

Bibliography

1. Gupta, R.; Weimer, W.A., [High enhancement factor gold films for surface enhanced Raman spectroscopy](https://www.sciencedirect.com/science/article/pii/S0009261403007371), *Chem. Phys. Lett*. **2003**, *374*, 302–306.
